# A Parametric Study on the Immunomodulatory Effects of Electroacupuncture in DNP-KLH Immunized Mice

**DOI:** 10.1093/ecam/nep166

**Published:** 2011-02-14

**Authors:** Sun Kwang Kim, Youngseop Lee, Hyunjoo Cho, Sungtae Koo, Sun Mi Choi, Min-Kyu Shin, Moo-Chang Hong, Byung-Il Min, Hyunsu Bae

**Affiliations:** ^1^Department of Physiology, College of Oriental Medicine, Kyung Hee University, Seoul 130-701, Republic of Korea; ^2^BK21 Oriental Medical Science Center, Kyung Hee University, Seoul 130-701, Republic of Korea; ^3^Acupuncture & Meridian Science Research Center, Kyung Hee University, Seoul 130-701, Republic of Korea; ^4^Department of Medical Research, Korea Institute of Oriental Medicine, Daejeon 305-811, Republic of Korea; ^5^Department of Meridian & Acupoint, School of Oriental Medicine, Busan National University, Busan 609-735, Republic of Korea; ^6^Department of Physiology, College of Medicine, Kyung Hee University, Seoul 130-701, Republic of Korea; ^7^Department of East-West Medicine, Graduate School, Kyung Hee University, Seoul 130-701, Republic of Korea

## Abstract

This study was conducted to compare the effects of low frequency electroacupuncture (EA) and high frequency EA at acupoint ST36 on the production of IgE and Th1/Th2 cytokines in BALB/c mice that had been immunized with 2,4-dinitrophenylated keyhole limpet protein (DNP-KLH), as well as to investigate the difference in the immunomodulatory effects exerted by EA stimulations at acupoint ST36 and at a non-acupoint (tail). Female BALB/c mice were divided into seven groups: normal (no treatments), IM (immunization only), ST36-PA (IM + plain acupuncture at ST36), ST36-LEA (IM + low frequency (1 Hz) EA at ST36), ST36-HEA (IM + high frequency (120 Hz) EA at ST36), NA-LEA (IM + low frequency (1 Hz) EA at non-acupoint) and NA-HEA (IM + high frequency (120 Hz) EA at non-acupoint). EA stimulation was performed daily for two weeks, and total IgE, DNP-KLH specific IgE, IL-4 and IFN-**γ** levels were measured at the end of the experiment. The results of this study showed that the IgE and IL-4 levels were significantly suppressed in the ST36-LEA and ST36-HEA groups, but not in the NA-LEA and NA-HEA groups. However, there was little difference in the immunomodulatory effects observed in the ST36-LEA and ST36-HEA groups. Taken together, these results suggest that EA stimulation-induced immunomodulation is not frequency dependent, but that it is acupoint specific.

## 1. Introduction

Acupuncture, which has been used for the treatment of various diseases in Eastern countries for thousands of years, is currently gaining acceptance as an alternative method of medicine in Western countries [1, 2]. Electroacupuncture (EA) is a modified acupuncture technique that utilizes electrical stimulation. Acupuncture or EA stimulation accelerates the release of certain neurotransmitters, especially opioids, noradrenalin (NA) and serotonin (5-HT), in the Central Nervous System (CNS), which then induces analgesia of acute and chronic pain [3–6]. In addition, several studies have suggested that EA stimulation at different frequencies and acupoints produces differing degrees of analgesia [7–9].

In addition to the analgesic effects of EA, several clinical studies have indicated that EA treatment is effective for the treatment of allergic disorders [10–12]. A limited number of animal experiments also have shown that EA stimulation can modulate immune response. For example, EA treatment upregulated NK cell activity in non-immunized mice and rats, and such effect was blocked by pretreatment with an opioid receptor antagonist, naloxone and by lesion of the lateral hypothalamus, respectively [13, 14]. In previous studies, we showed that successive EA treatment reduced serum IgE levels in mice immunized with 2,4-dinitrophenylated keyhole limpet protein (DNP-KLH) through the suppression of the Th2 cytokines [15], and that this effect was reversed by phentolamine (*α*-adrenoceptor antagonist) pretreatment, but not by naloxone pretreatment [16]. However, few parametric studies regarding EA stimulation-induced immunomodulation have been conducted, suggesting that the evaluation of the immunomodulatory effects of EA under different stimulation conditions is required. Therefore, in this study, we determined whether the modulatory effects of EA on immune response in DNP-KLH immunized mice are dependent on the frequency of electrical stimulation or stimulation of a specific accupoint. To accomplish this, we compared the effects of low frequency (1 Hz) and high frequency (120 Hz) EA at the ST36 acupoint on IgE and cytokines in immunized mice, as well as examined the difference in the immunomodulatory effects of ST36 acupoint and non-acupoint (tail) EA stimulations.

## 2. Methods

### 2.1. Animals

Female BALB/c mice (8 weeks, OrientBio, Korea) were housed in an air-controlled, pathogen-free animal facility with a 12-h light/dark cycle at 23 ± 2°C. Food and water were available *ad libitum*. All of the procedures involving animals were approved by the Institutional Animal Care and Use Committee at Kyung Hee University.

### 2.2. Experimental Groups

Mice were randomly divided into seven groups: (i) Normal group (no treatments, with the exception of an i.p. injection of saline on the first and eighth experimental day, *n* = 6); (ii) IM group (immunization on the first and eighth day, *n* = 8); (iii) ST36-PA group (immunization + daily plain acupuncture (needle insertion only with no manipulation) at ST36, *n* = 8); (iv) ST36-LEA group (immunization + daily low frequency EA at ST36, *n* = 10); (v) ST36-HEA group (immunization + daily high frequency EA at ST36, *n* = 8); (vi) NA-LEA group (immunization + daily low frequency EA at non-acupoint, *n* = 6); (vii) NA-HEA group (immunization + daily high frequency EA at non-acupoint, *n* = 6). All mice, with the exception of those in the normal group, were immunized intraperitoneally with 4 *μ*g of DNP-KLH (Calbiochem, San Diego, CA, USA) and 4 mg of aluminum hydroxide (Sigma, USA) on the first and eighth day during the 2-week experimental period [15, 16].

### 2.3. EA Stimulation

EA stimulation was performed as described previously [15, 16]. Briefly, a pair of stainless steel needles (0.2 mm in diameter and 3 cm long) were inserted (3 mm in depth) into the ST36 acupoint, which is located at the anterior tibial muscle, 5 mm laterally and lower from the anterior tubercle of the tibia, and at a point 5 mm distal from the first needle. EA stimulation at this point (ST36) is known to modulate the immune response in mice [14–16]. Anode and cathode leads from an electrical stimulator were connected to the two acupuncture needles, and then train-pulses (1 Hz (low frequency) or 120 Hz (high frequency), 0.25 ms pulse width, 3–5 v) were applied for 20 min. The mice in the NA-HEA and NA-LEA group were subjected to the same EA stimulation at a non-acupoint, which is located on the proximal part of the tail [17].

### 2.4. Assays of IgE and Cytokines

Serum and splenocytes from all of the mice were obtained on the final experimental day. The levels of total and antigen-specific IgE in the serum, as well as the amounts of IL-4 and IFN-*γ* from anti-CD3 mAb-activated splenocytes were measured by Enzyme-linked immunosorbent assay (ELISA) using a method that has been described previously [15].

### 2.5. Statistics

For the statistical analysis, the one-way analysis of variance (ANOVA) followed by Newman-Keuls multiple comparison test was used. Data were presented as the mean ± SEM. In all cases, *P* <  .05 was considered to be significant. Each of the EA stimulation, cytokine assays and statistical analysis was performed in an evaluator-blinded fashion.

## 3. Results

### 3.1. EA Treatments at ST36 Acupoint, but not at Non-Acupoint, Reduced Serum IgE Levels Irrespective of the Stimulation Frequency

**Figure 1:**
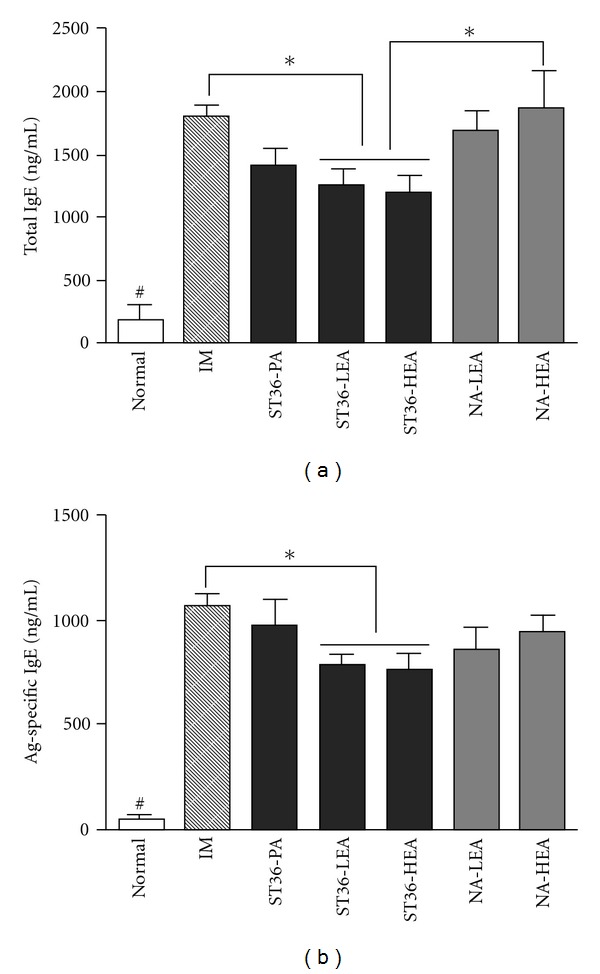
Effects of EA using different parameters on serum levels of total and antigen-specific IgE in DNP-KLH immunized mice. The amounts of total IgE (a) and antigen-specific IgE (b) were measured by the ELISA. Data were presented as the mean ± SEM. *P <  .05, between the indicated two groups; ^#^ *P* <  .001, Normal versus all other groups by Newman-Keuls multiple comparison test after one-way ANOVA. Normal, no treatments; IM, immunization; ST36-PA, immunization + plain acupuncture at ST36; ST36-LEA, immunization + low frequency (1 Hz) EA at ST36; ST36-HEA, immunization + high frequency (120 Hz) EA at ST36; NA-LEA, immunization + low frequency EA at non-acupoint; NA-HEA, immunization + high frequency EA at non-acupoint.

The total IgE and antigen-specific IgE levels in the serum are shown in Figure 1. Immunization with DNP-KLH significantly increased both the total and antigen-specific IgE levels (*P* <  .001, Normal versus IM), and this increase was significantly attenuated by low or high frequency EA stimulation of the ST36 acupoint (*P* <  .05, IM versus ST36-LEA or ST36-HEA). However, the suppressive effects of EA stimulation on the IgE levels were not different between the ST36-LEA and ST36-HEA groups (*P* >  .05). In addition, the total and antigen-specific IgE levels in the ST36-PA group were not significantly different from those of the IM group or the ST36 EA-treated groups (*P* >  .05, ST36-PA versus IM, ST36-LEA or ST36-HEA). The results from the NA-LEA group were similar to the ST36-PA group (*P* >  .05, NA-LEA versus IM, ST36-LEA or ST36-HEA). Additionally, high frequency EA at the non-acupoint did not affect the total IgE levels (*P* >  .05, NA-HEA versus IM; *P* <  .05, NA-HEA versus ST36-LEA or ST36-HEA), however, slightly reduced antigen-specific IgE levels similar to those observed in the ST36-PA and NA-LEA group were observed in the NA-HEA group (*P* >  .05, NA-HEA versus IM, ST36-LEA or ST36-HEA).

### 3.2. ST36 EA Markedly Suppressed IL-4 Levels in the Splenocytes with No Difference between Low- and High-Frequency Stimulation

**Figure 2:**
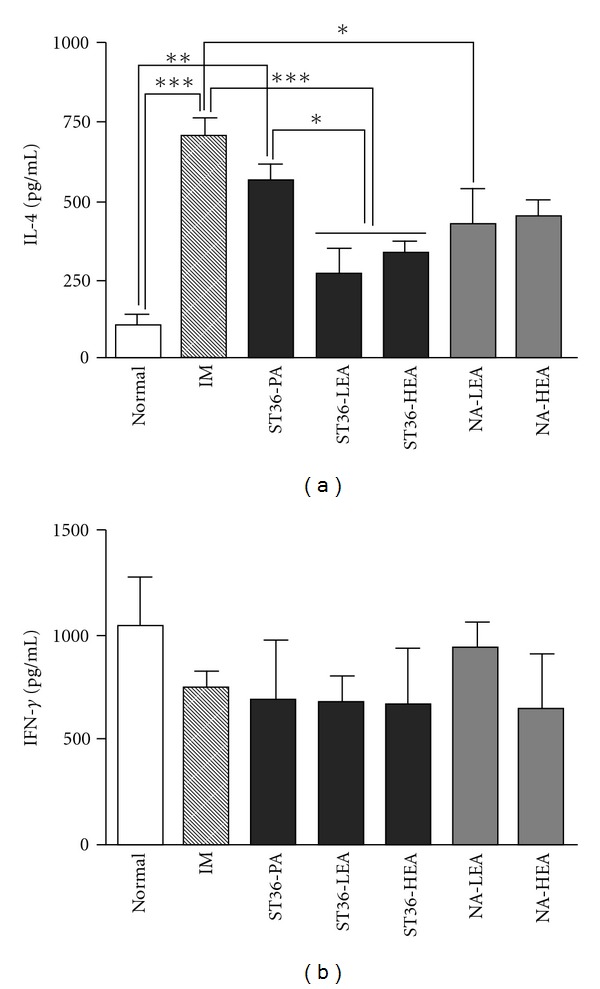
Effects of EA using different parameters on IL-4 and IFN-*γ* secretion from splenocytes in DNP-KLH immunized mice. The amounts of IL-4 (a) and IFN-*γ* (b) were determined by the ELISA. Data were presented as the mean ± SEM. *P <  .05, **P <  .01, ***P <  .001, between the two indicated groups as determined by Newman-Keuls multiple comparison test after one-way ANOVA. Normal, no treatments; IM, immunization; ST36-PA, immunization + plain acupuncture at ST36; ST36-LEA, immunization + low frequency (1 Hz) EA at ST36; ST36-HEA, immunization + high frequency (120 Hz) EA at ST36; NA-LEA, immunization + low frequency EA at non-acupoint; NA-HEA, immunization + high frequency EA at non-acupoint.

The levels of IL-4 and IFN-*γ* from the splenocytes are shown in Figure 2. Similar to the results of previous studies [15, 16, 18], DNP-KLH immunization caused a marked increase in IL-4 secretion (*P* <  .001, IM versus Normal), whereas it only slightly suppressed IFN-*γ* levels (*P* >  .05, IM versus Normal). However, both low and high frequency EA at ST36 significantly inhibited this increase in IL-4 (*P* <  .001, ST36-LEA or ST36-HEA versus IM), although EA at either frequency had no effect on IFN-*γ* secretion (*P* >  .05, ST36-LEA or ST36-HEA versus IM). In addition, there was little difference in the IL-4 and IFN-*γ* levels observed in the ST36-LEA and ST36-HEA groups (*P* >  .05). Plain acupuncture at ST36 had no effect on the IL-4 levels (*P* >  .05, ST36-PA versus IM; *P* <  .01, ST36-PA versus Normal; *P* <  .05, ST36-PA versus ST36-LEA or ST36-HEA) or the IFN-*γ* levels (*P* >  .05, ST36-PA versus IM). Interestingly, low frequency EA at the non-acupoint significantly inhibited IL-4 production (*P* <  .05, NA-LEA versus IM). Low frequency EA at the non-accupoint also slightly upregulated IFN-*γ*, however, this change was not statistically significant (*P* >  .05, NA-LEA versus all other groups). Finally, the NA-HEA group showed a slight reduction in IL-4 production, but this reduction was not statistically significant (*P* >  .05, versus all other groups) and there was also no change observed in the IFN-*γ* levels of this group (*P* >  .05, versus IM).

## 4. Discussion

To the best of our knowledge, this study represents the first parametric study of the immunomodulatory effects of EA. In this study, we evaluated EA-stimulation-induced immunomodulation in DNP-KLH immunized mice to determine if it was dependent upon three parameters: (i) electrical stimulation (plain acupuncture versus EA); (ii) frequency (low frequency versus high frequency EA) and (iii) acupoint (ST36 acupoint versus non-acupoint).

The data obtained here showed that the EA stimulation of ST36, but not ST36 plain acupuncture, significantly inhibited the elevation of IgE and IL-4 levels that were induced by immunization, which indicates that electrical stimulation is necessary for a significant immunomodulatory effect of acupuncture at ST36 in DNP-KLH immunized mice to occur. This result is consistent with the results of previous studies that have shown that electrical stimulation or manipulation, such as rotation and varying the depth of needle insertion, is required to increase the analgesic effects of plain acupuncture in animals and humans [8, 9, 19, 20].

It is well known that EA stimulation using different frequencies produces differing degrees of analgesia, which is mediated by a different mechanism [4–9, 21]. However, the responses to different frequencies of EA stimulation vary. For example, Lin et al. [9] reported that high frequency EA provided better results than low frequency EA at reducing the postoperative analgesic requirements and opioid-related side-effects following lower abdominal surgery. Additionally, Lao et al. [8] showed that high-frequency EA produced potent and shorter lasting analgesic effects, whereas low-frequency EA had moderate and longer-lasting effects on CFA-induced hyperalgesia in rats. Conversely, our previous studies [6, 21] showed that low-frequency EA produced more robust and longer lasting effects on neuropathic pain in rats than high-frequency EA. Taken together, these results suggest that low-frequency and high-frequency EA have different efficacies on analgesia, depending on experimental conditions. Therefore, we expected low- and high-frequency EA treatments to induce different immunomodulatory effects, even under our experimental condition (Th2 dominant condition). However, little difference in the effects on IgE hyperproduction and IL-4 upregulation in DNP-KLH immunized mice was observed between the ST36-LEA and ST36-HEA groups. This finding suggests that EA-stimulation-induced immunomodulation is not dependent on frequency in DNP-KLH immunized mice.

Although contradictory results regarding accupoint specificity have been reported, a number of animal studies have shown that the EA effects are acupoint specific. Studies using various types of disease models have indicated that EA stimulation performed elsewhere on the body does not produce the same effect and does not involve the same mechanism as stimulation at a specific acupoint, such as ST36 [17, 22–24]. In order to examine the specific acupoint effect, the NA-LEA and NA-HEA groups in the present study were used as active controls for the ST36-LEA and ST36-HEA groups. Although a significant reduction in IL-4 levels was observed in the NA-LEA group (Figure 2(a)), the results of this study suggest that non-acupoint EA stimulation, irrespective of frequency, has only a slight effect on immune responses in DNP-KLH immunized mice.

In conclusion, our parametric study demonstrated that the immunomodulatory effects of EA in DNP-KLH immunized mice are not frequency dependent, but that they are acupoint specific. Because the present experimental condition (Th2 skewed immune response) represents only one of the probable situations related to diverse immune disorders, further parametric studies using other models are needed for the effects of EA using different stimulation conditions on immune responses to be elucidated.

## Funding

This work was supported by the Acupuncture, Moxibustion & Meridian Research Project of the Korea Institute of Oriental Medicine in 2007. H.B. was partly supported by the SRC program (R11-2005-014) of KOSEF, Korea.
